# Lymphatic Filariasis Increases Tissue Compressibility and Extracellular Fluid in Lower Limbs of Asymptomatic Young People in Central Myanmar

**DOI:** 10.3390/tropicalmed2040050

**Published:** 2017-09-27

**Authors:** Janet Douglass, Patricia Graves, Daniel Lindsay, Luke Becker, Maureen Roineau, Jesse Masson, Ni Ni Aye, San San Win, Tint Wai, Yi Yi Win, Susan Gordon

**Affiliations:** 1 Division of Tropical Health and Medicine, James Cook University, Townsville 4811, Australia; daniel.lindsay1@jcu.edu.au (D.L.); sue.gordon@flinders.edu.au (S.G.); 2 Division of Tropical Health and Medicine, James Cook University, Cairns 4870, Australia; patricia.graves@jcu.edu.au (P.G.); luke.becker@jcu.edu.au (L.B.); maureenroineau@wanadoo.fr (M.R.); jesse.masson@my.jcu.edu.au (J.M.); 3 Department of Health, Myanmar Ministry of Health and Sports, Nay Pyi Taw 15011, Myanmar; shwewaethu@gmail.com (N.N.A.); mr.tintwaitun2013@gmail.com (T.W.); yywin2008@gmail.com (Y.Y.W.); 4 World Health Organization, Country Office, Yangon 11201, Myanmar; wins@who.int; 5 College of Nursing & Health Sciences, Flinders University, Bedford Park 5042, Australia

**Keywords:** neglected tropical disease, lower extremity, lymphatic filariasis, tissue tonometry, bio-impedance spectroscopy, lymphedema

## Abstract

When normal lymphatic function is hampered, imperceptible subcutaneous edema can develop and progress to overt lymphedema. Low-cost reliable devices for objective assessment of lymphedema are well accepted in clinical practice and research on breast-cancer related lymphedema but are untested in populations with lymphatic filariasis (LF). This is a cross-sectional analysis of baseline data in a longitudinal study on asymptomatic, LF antigen-positive and -negative young people in Myanmar. Rapid field screening was used to identify antigen-positive cases and a group of antigen-negative controls of similar age and gender were invited to continue in the study. Tissue compressibility was assessed with three tissue tonometers, and free fluids were assessed using bio-impedance spectroscopy (BIS). Infection status was confirmed by Og4C3 antigen assay. At baseline (*n* = 98), antigen-positive cases had clinically relevant increases in tissue compressibility at the calf using a digital Indurometer (11.1%, *p* = 0.021), and in whole-leg free fluid using BIS (9.2%, *p* = 0.053). Regression analysis for moderating factors (age, gender, hydration) reinforced the between-infection group differences. Results demonstrate that sub-clinical changes associated with infection can be detected in asymptomatic cases. Further exploration of these low-cost devices in clinical and research settings on filariasis-related lymphedema are warranted.

## 1. Introduction

Lymphatic filariasis (LF) is a parasitic disease in which thread-like worms inhabit the human lymphatic system, where they can impair normal lymphatic pumping. Classified as a neglected tropical disease and affecting many of the world’s poorest populations, LF can lead to lymphedema, a progressively debilitating swelling of the skin and subcutaneous tissue in any body part, most frequently the legs [1]. Normal lymphatic pumping actively removes circulating proteins and fluid from the tissue spaces, maintaining a slightly negative interstitial pressure. When lymphatic capacity is impaired, extracellular fluid (ECF) and circulating proteins begin to accumulate in the interstitial spaces [2]. If normal lymphatic function is not restored, this initially covert edema gradually becomes overt, and the affected body part visibly enlarges. Over time, the protein-rich fluid is replaced with fat and fibrous tissue, and normal limb contours are lost. The outdated eponym ‘elephantiasis’ was inspired by the appearance of a grossly enlarged limb in late-stage lymphedema where the skin is thick, discolored, and formed into folds. In developed countries, lymphedema is frequently caused by surgical damage when lymph nodes are removed or irradiated during cancer treatment. Much of what is known about initiation and progression of lymphedema comes from research on breast cancer-related lymphedema (BCRL) of the arm [3].

A wide spectrum of devices and methods is used to objectively evaluate lymphedema depending on the setting. At the highly resourced end of the spectrum, nuclear imaging and other sophisticated technologies are often used to assess BCRL of the arm. Tissue tonometry to quantify tissue compressibility and portable bio-impedance spectroscopy (BIS) to track fluctuations in free fluid are also used and are relatively inexpensive. Using BIS, it has been shown that covert pathologic change due to lymphatic damage during breast cancer treatment can be detected, and that early intervention in this latent stage can prevent the onset of overt disease [4]. At the low-resourced end of the spectrum, assessment of LF related-lymphedema (LFRL) of the leg usually relies on classification of visible and palpable soft tissue changes [5], where subjectivity may lead to inconsistent classification. There is no differentiation or assessment of covert change, so subtle but important alterations in tissue composition may be missed.

In LF, mosquitoes pick up the microfilariae during a blood meal. The larvae develop to third stage within the mosquito before being transmitted by a subsequent bite. Transmission is relatively inefficient with a low risk of infection per bite, and after transmission there is a lag between being infected and the development of adult worms. This means that most children with LF will remain asymptomatic until young adulthood, which affords a long, latent period in which to implement preventive strategies [6]. Primary prevention in the Global Program to Eliminate LF (GPELF) is preventive chemotherapy, which is delivered annually via mass drug administration (MDA) in endemic regions [7]. This will eventually prevent any new cases of morbidity as infection rates fall too low to sustain transmission. However, preventive chemotherapy conveys no real benefit to advanced cases, most of whom will no longer be antigenemic, but will require life-long health care. In between the asymptomatic cases that will never progress to overt disease and the advanced cases that have irreversible lymphedema, there are many cases of latent and early stage lymphedema. There is some evidence that MDA may reverse very early tissue changes in LFRL [8], but without standardized assessment or diagnostic criteria for Stage 0, or devices sensitive enough to detect small changes in tissue composition, it is not clear at what stage or which individuals will remain at risk of disease progression. Reliable, sensitive, low-cost devices to provide objective assessment of LFRL are needed [9].

A pilot study in Papua New Guinea (PNG) found the skin over the posterior thigh was 20% more compressible in asymptomatic young people who had tested positive for LF antigen compared to antigen-negative peers, using a mechanical tonometer [10]. Subsequently, three tissue tonometers and a portable BIS device have demonstrated intra-operator reliability in assessing tissue composition in the lower limbs of young Australian and Myanmar populations without any history or risk of lymphedema [11]. It is not yet known if covert lymphedema can be detected by tissue tonometry or BIS in these populations.

There is no agreed standard for assessment of Stage 0 lymphedema, and diagnostic criteria for clinical onset are not well defined [3]. One study on BCRL used a 3% change in BIS values to trigger preventive treatment [4], and clinical lymphologists may use a percentage change in limb girth or volume to track lymphedema change, with a variation of more than 10% considered clinically relevant [12]. Variations in body composition will influence measurements with these devices as muscle holds more free fluid than fat, fat is more compressible than muscle, and the ECF in the subcutaneous compartment fluctuates slightly depending on overall body hydration. Individual characteristics that influence body composition should be considered when assessing superficial tissues of the lower limb, including expected changes associated with growth from child to young adult and gender-based differences in muscle and fat distribution. Habitual patterns of muscle use should also be considered, and significant between-leg differences in healthy young Australian and Myanmar people have been reported when using these devices [13].

This cross-sectional study on young people residing in an LF endemic region in Central Myanmar investigated whether tissue tonometry and BIS measures were altered in asymptomatic cases who tested positive for *Wuchereria bancrofti* antigen. The results will assist researchers and clinicians to objectively quantify changes occurring in early LFRL and may contribute to formal recognition and intervention for Stage 0 lymphedema of the leg.

## 2. Materials and Methods

### 2.1. Study Site Selection, Participant Recruitment, and Screening

Sentinel site records kept by the Vector Borne Disease Control (VBDC) Centre in Mandalay identified Amarapura Township as a densely populated area with a high prevalence of LF. It was also close enough to laboratory services for blood sample processing. A study site was set up in the Administration Centre in the village of Nge Toe and baseline data were collected over a two-week period in October 2014. The study was conducted in accordance with the Declaration of Helsinki and the protocol was approved by the Myanmar Ministry of Health (MoH) and James Cook University Human Research Ethics Committee (approval number H5261).

**Figure 1:**
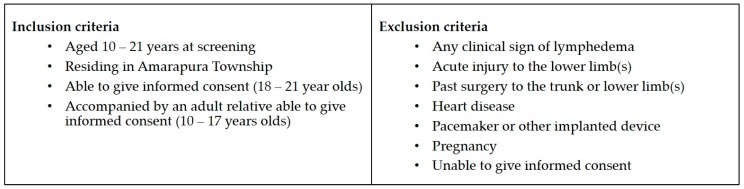
Participant inclusion and exclusion criteria.

A sample size of 32 in each group was predicted to detect a 10% difference between groups with 80% power, based on a mean mid-calf value of 2.5 with SD of 0.7 using the digital Indurometer [13,14]. A convenience sample of local young people aged 10–21 years was invited to be screened for LF antigen and to participate in a longitudinal study on early detection of LFRL. Participant information sheets and informed consent forms were provided in Burmese. Staff of the VBDC and Amarapura Township Hospital, the World Health Organization (WHO) technical officer for Myanmar (SSW), and locally-trained research assistants explained all procedures to the participants, determined their eligibility to participate, and obtained informed consent. Written consent was given by young adults aged 18–21 and by a parent or guardian for minors aged 10–17. A further verbal assent for each procedure was obtained from all participants prior to performing that procedure. Participant inclusion and exclusion criteria are shown in Figure 1.

### 2.2. Screening and Baseline Data Collection

A rapid field test for the presence of LF antigen was performed using an immunochromatographic test (ICT) card (Binax Now, Alere, Waltham, MA, USA). This involved placing a 100 µL draw of blood from a fingerprick onto a test strip. The sample was allowed to flow for 10 min and the result appeared as one or two lines across the test strip. One line is a control and if this line was not visible then the test was void and if possible, repeated. Appearance of the second line indicated the presence of circulating *W. bancrofti* antigen that is produced by adult worms. The young people who tested positive by ICT (cases), and a sample of the negative participants of the same age and gender (controls), were invited to return and participate in the longitudinal study. A James Cook University (JCU) technical staff member (LB) trained the local research assistants in correct use of the ICT card and selected participants invited for follow-up.

Participants returned during the following fortnight for the blood draw and device measures. Local research assistants conducted a short interview to elicit information on current health status, prescription or traditional medications, surgical history, family history of lymphedema, time since the last drink (as a proxy for hydration), and if they had consumed preventive chemotherapy during the previous annual MDA. Leg dominance was determined by asking the question ‘Which foot do you use to kick a ball?’ Height was measured using a chart marked on a wooden post in centimeters and a set square, and weight in kilograms was recorded using digital scales purchased locally. Device measures were conducted in a small side office or screened off area and an adult relative was asked to be present during the measurement of minors.

#### 2.2.1. Device Measures

Three tissue tonometers were used to assess tissue compressibility. The Indurometer (SA Biomedical Engineering, Adelaide, Australia) is a hand-held electro-mechanical device with a 1 cm diameter plunger/indenter extending through a 7 cm diameter reference plate and a built-in force sensor. The reference plate is aligned to the surface of the skin while the device is pressed evenly into the tissue. A beep is emitted once the equivalent to 200 g of force has been applied, and the degree of displacement is displayed in 0.01 increments on a light-emitting diode (LED) screen. An image of the Indurometer is shown in Figure S1. The mechanical Tonometer (SA Biomedical Engineering, Adelaide, Australia) is a similar device, in which a 1 cm diameter plunger extends beyond a 7 cm diameter reference plate. This purely mechanical device uses a 200 g mass to drive the indenter into the underlying tissue, and the degree of displacement is shown on an analogue scale. Both of these devices record the displacement of the indenter in relation to the reference plate as an indication of compliance (compressibility) of the underlying skin and tissue. The values provided by these devices are not absolute measures and can be considered as arbitrary units used to compare measures of tissue compressibility [15]. A third device, the SkinFibroMeter (Delfin Technologies, Kuopio, Finland), uses a smaller reference plate with a 1.25 mm length fixed indenter and built-in force sensors. The reference plate is pressed evenly onto the skin and the device emits a beep when the equivalent of 50 g has been applied. The device is applied five times and the average resistance in newtons is displayed on a digital screen. A tape measure and washable skin marker were used to locate and mark the midpoint of each thigh (front and back) and the back of each calf, and all tonometry measures were taken at these marks.

Extracellular and intracellular fluid loads were assessed using bio-impedance spectroscopy (BIS), which measures the resistance to multifrequency, low-level electrical currents. The difference between resistance in the intracellular (Ri) and extracellular (Re) fluid compartments was represented as a ratio Ri:Re. As the intracellular fluid (ICF) compartment is tightly regulated, any changes in the ratio usually represent changes in the extracellular fluid (ECF). Whole-leg BIS measures were recorded for each leg with the SFB7 (Impedimed, Australia) using self-adhesive electrodes applied to the skin according to manufacturer’s instructions for lower limb measures.

A detailed description of data collection methods was published in a reliability study on these devices in Australia and Myanmar [11]. All devices were operated by the principal researcher (JD), who was blinded to the infection status of the participants. Tonometry scores were recorded on data collection sheets by a research assistant, and BIS data was downloaded to an Excel file (Microsoft Office 365, version 1706).

#### 2.2.2. Blood Collection and Processing/Storage

Blood samples were collected by local research assistants, who were trained in specific blood collection and handling protocols by the JCU technician (LB). A 10 mL draw of venous blood was collected from each participant into cooled ethylenediaminetetraacetic acid (EDTA) anticoagulant vacutainers (BD Biosciences, North Ryde, Australia). The antigen test was repeated using 100 uL of the venous blood pipetted onto an ICT card, and the remaining blood was kept on ice until delivery to the Public Health Laboratory in Mandalay. Separation of plasma and red blood cells was performed using a centrifuge for 15 min at 3000 rpm; the plasma was transferred into 2-mL cryotubes by pipette in duplicate (4 mL per person) and stored at −20 °C. Once all baseline data had been collected, the plasma was transferred on dry ice to the Department of Medical Research in Yangon for long-term storage at −80 °C in a monitored freezer connected to a back-up generator and with daily monitoring. There were no thaws during plasma transportation or storage. One set of the cryotubes was aliquoted and used to conduct ELISA assay for the presence of Og4C3, an antigen marker for *W. bancrofti*, using the recommend 1:4 dilution for plasma as per the manufacturer (Cellabs, Sydney, Australia) kit instructions [16]. Samples were classified as positive if the antigen units, estimated using the standard curve of controls provided with the kit, exceeded 32 units. Detailed methods for the ELISA assays were previously published in a study on diagnostic testing for LF antigen [16].

### 2.3. Data Analysis

LF antigen-positive cases were defined as those who were positive by either antigen test (ICT or Og4C3). Body mass index (BMI) was calculated as kg/m^2^, but adult values cannot be used for children; therefore, WHO growth charts and definitions were used to identify underweight participants, who were defined as being more than two standard deviations below the median BMI for their age [17]. Chi-squared tests, Fisher’s exact tests, and independent samples *t*-tests were used to compare antigen-positive and -negative group characteristics at baseline for known moderating factors. Paired sample *t*-tests were used to compare device measures of dominant and non-dominant legs. Statistical analysis was conducted in SPSS version 23 (IBM Corp), and significance was set at 0.05 with a 95% confidence interval. Clinically-relevant difference for tonometry measures was set at >10% and for BIS measures it was set at >3%. Stepwise regression was performed for dominant and non-dominant legs separately to determine the level of variance in device measures associated with infection status (univariate) and other potential moderating factors (multivariate).

## 3. Results

### 3.1. Participants

**Figure 2:**
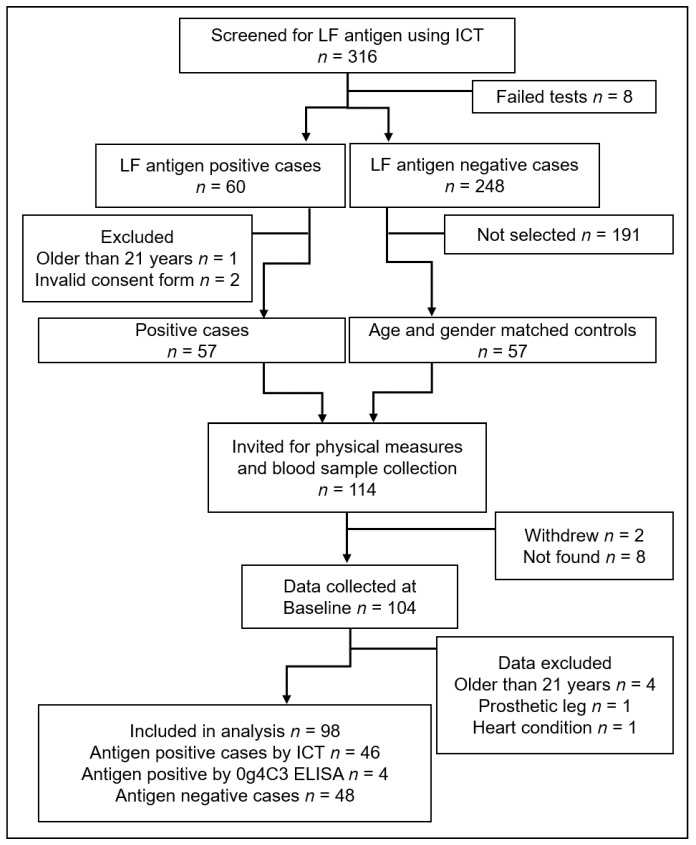
Flow of participants through recruitment, screening, and baseline data collection.

Screening for LF found 60 antigen-positive cases among 316 volunteers, and 114 young people (57 cases and 57 controls of the same age and gender) were invited to continue in the longitudinal study (see Figure 2). Ten people either could not be found or refused to return, and 104 participants were available for baseline blood draw and physical measures. Data from six participants were excluded from the final analysis; four were found at a later measure to have been outside the target age range at baseline, one had a prosthetic leg, and another had a heart condition, neither of which had been disclosed at the screening interview. The final study population was comprised of 46 antigen-positive cases detected by ICT plus a further four cases identified as antigen positive by Og4C3 ELISA (*n* = 50). There were 48 antigen-negative (control) cases.

#### 3.1.1. Participant Characteristics

All participants (*n* = 98) were aged between 10 and 21 years (mean 15.3 SD 3.4) and there were 55 females and 43 males. The mean height, weight, and BMI were 152.0 cm (SD 12.0, range 118.8–174.0), 42.3 kg (SD 11.5, range 17.5–82.7), and 18.0 kg/m^2^ (SD 3.0, range 12.4–29.7), respectively. The cohort was 95.9% right leg dominant and 13.3% (*n* = 13) were considered underweight. Almost half (44.9%) of the participants were working in weaving workshops, 27.6% were students, 8.2% were street vendors, 2.0% were construction workers, and the remaining 17.3% worked in other occupations or did not disclose their occupation. None had a history of lymphedema in their immediate family, previous surgery or medical implants, and all were in good health. Two participants were taking prescription medications and one was using traditional medicine. One participant felt unwell on the day scheduled for taking the measures and was asked to return when feeling better. Comparing antigen-positive and antigen-negative groups, there were no significant between-group differences for any physical attribute or moderating factor. Participant characteristics at baseline are shown in Table 1.

### 3.2. Moderating Factors Associated with Device Measures

#### 3.2.1. Effect of Infection on Device Measures

In the antigen-positive group, tissue compressibility was higher at all measuring points, and there was more free fluid in both legs compared to that of the antigen negative group. Independent *t*-tests found that, at mid-calf on the non-dominant side, the increase in Indurometer measures was both clinically (11.1%) and statistically significant (*p* = 0.021). In addition, whole leg BIS measures found clinically-relevant (>3%) increases in free fluid in both legs (dominant leg, 4.9% (*p* = 0.220), non-dominant leg, 9.2% (*p* = 0.053)). Mean values and between-group differences for the Indurometer and BIS measures are shown in Table 2. Neither the mechanical Tonometer nor SkinFibroMeter found any clinically relevant or statistically significant differences between infection groups, with many differences too small to be evident at two decimal places. The only between-group difference of interest with these two devices was increased tissue compressibility with the Tonometer at the non-dominant calf (4.8% softer, *p* = 0.296). Mean values and between-group differences for all devices including the Tonometer and SkinFibroMeter are given in Table S1.

#### 3.2.2. Effect of Infection Status, Age, Gender, Body Composition, and Hydration on Device Measures

Regression was first performed with infection status (antigen positivity) alone, and then stepwise regression was used to add moderating factors. Being antigen positive was significantly associated with increased compressibility in the non-dominant calf when using the Indurometer (Table 3, step 1) which is consistent with the *t*-test results given above in Table 2. Using multivariate regression, after adjustment for other factors (gender, age, underweight, and hydration), increased compressibility remained significantly associated with being antigen positive (in the non-dominant calf) using the Indurometer, and was also significant in the dominant calf using the same device. When considering all factors, being antigen positive was significantly associated with increased fluid in the non-dominant leg using BIS (Table 3, step 2).

In the stepwise regression, being female was significantly associated with higher tissue compressibility using all three tonometers. The largest gender-related effect using the Indurometer was in calf measures where there is a relatively thin fat layer over the muscles, making small differences in fat and muscle composition more likely to be detected (dominant leg B (SE) = 0.639 (0.117), *p* < 0.000) (see Table 3). The least effect of gender was found over the anterior thighs where the relatively thicker fat layer reduces the influence of the underlying muscle tone and a small difference between the sexes is not likely to register as much change. Using BIS, being female was significantly associated with less free fluid in both legs, and this is consistent with females having relatively smaller muscle/higher fat mass (less fluid) than males of the same age. The largest coefficient was in the non-dominant leg (B (SE) = 0.485 (0.103), *p* < 0.001) (see Table 3).

Being less well hydrated, defined as not having a drink within one hour of measures, was associated with lower tissue compressibility. This was significant at the non-dominant calf (B (SE) = −0.239 (0.110), *p* = 0.032). Being older was significantly associated with a small increase in free fluid in both legs, consistent with normal growth increase in muscle mass. Being underweight was significantly associated with a small increase in free fluid in the non-dominant leg (BIS) which may be associated with reduced fat mass or an increased capillary filtrate due to proteinemia.

In summary, when accounting for known moderating factors of age, gender, BMI, and hydration, there was a highly significant association between antigen positivity and increased Indurometer measures at the non-dominant calf (*p* = 0.007). At the dominant calf, the same association was also significant (*p* = 0.038). When these factors are taken into account for BIS measures, there was a clinically relevant and significant increase in free fluid (Table 2) in the non-dominant leg (*p* = 0.038). All associations between moderating factors and device measures are given in Table S2.

### 3.3. Patterns of Tissue Compressibility and Free Fluid in Dominant and Non-Dominant Legs

There was a consistent pattern of tissue compressibility at the measurement sites that held true for all devices and all subgroups by age, gender, or infection status. The most compressible tissue was located at the (relatively) fatty anterior thigh, the least compressible tissue was over the dense tendomuscular junction at mid-calf, and values for the posterior thigh fell between the two. When comparing dominant and non-dominant legs, a consistent pattern of between-leg differences was seen and can be attributed to expected muscle activity during a kick. The skin was less compressible (more muscle tone) over the front of the ‘dominant’ kicking thigh and over the back of the ‘non-dominant’ thigh and calf muscles that propel the body forward during the kick. Using BIS, there was more free fluid (more muscle mass or less fat) in the dominant leg compared to the non-dominant leg (9.6%); this difference was both clinically relevant (>3%) and statistically significant (*p* < 0.01) using paired samples *t*-tests. Mean values and between-leg differences for the Indurometer and BIS are given in Table 4. Mean values and between-leg differences for all devices including the Tonometer and SkinFibroMeter and are given in Table S3.

**Figure 3:**
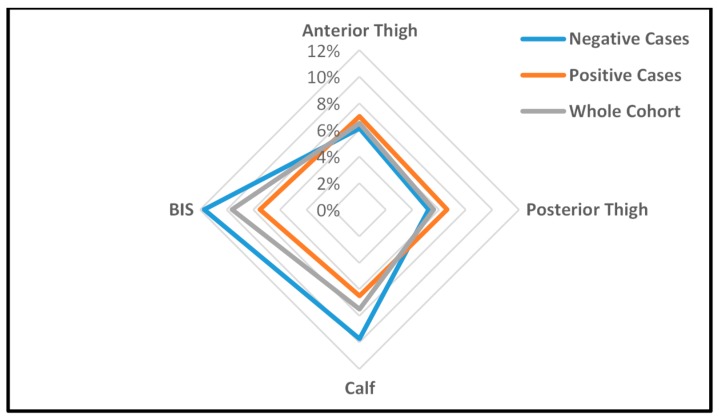
Percentage between-leg differences using the Indurometer and BIS in the LF antigen-negative cases, LF antigen-positive cases, and whole cohort. Data points which are closer to the outer ring indicate greater between-leg differences.

The overall pattern of between-leg differences (dominant vs. non-dominant), as demonstrated by kicking a ball, was maintained in the antigen-positive cases, but the degree of difference was altered. Figure 3 is a radar graph showing the percentage of between-leg differences in Indurometer and BIS values for the whole cohort and by infection group. In the infected group, between-thigh differences in tissue compressibility were exaggerated (closer to the outer ring in the radar chart) but only slightly, with similar percentage differences for positive (7%), negative (6.1%), and whole cohort groups (6.5%). The between-infection group differences were more pronounced at the calf where the mean between-calf difference in the positive cases (6.5%) was much smaller (closer to the middle) than that of the negative cases (9.7%) or whole cohort (7.5%). Similarly, as well as an overall increase in free fluid, BIS results indicated that positive cases had smaller between-leg differences compared to those of their negative counterparts (7.5% vs. 11.7%). Although not statistically significant, these reduced between-leg differences in the distal legs of the antigen-positive cases suggest a covert edema overlying and masking normal between-leg variations in muscle tone and mass.

## 4. Discussion

In this study, tissue compressibility and free fluid loads were higher in asymptomatic young people infected with LF compared to their uninfected peers. Both groups displayed normal patterns of within-leg tissue compressibility; i.e., tissue was most compressible over the anterior thigh and least compressible at the calf, and between-leg differences were consistent with kicking a ball. However, when stratified by infection status, the size and direction of between-leg differences in the positive cases were consistent with a covert accumulation of subcutaneous fluid in the lower leg. Usually, LFRL appears distally and progresses proximally, so detectable tissue changes may occur earlier at the calf than at the thigh. The relatively thin layer of skin and tissue over the muscle of the calf may also render early tissue changes more evident than in fattier parts of the leg. Accordingly, the association with LF antigenemia and Indurometer measures was statistically significant at mid-calf, and large enough on the non-dominant side to also be clinically relevant. This early appearance of lymphatic dysfunction in the non-dominant leg is consistent with reports on BCRL, which show an increased risk of arm lymphedema if the operated side is also the non-dominant arm [18]. This tendency for fluid to accumulate more readily on the non-dominant side could be the result of differences in muscular activity that naturally promotes lymph flow and may be greater or more frequent on the dominant side.

For all devices, the significant associations between higher tissue compressibility and lower free fluid in females reflect expected variation in muscle to fat ratios between the sexes. Other moderating factors such as hydration, although not as universal as gender, did have significant associations with measures at the calf, but this could be reduced by administering a standardized drink during the assessment protocol. Increased free fluid associated with age and being underweight can be attributed to a year-by-year increase in muscle mass, or a systemic reduction in fat mass, respectively.

Results in the Myanmar study reinforce earlier findings from PNG [10], where clinically significant between-infection group differences were found in physical leg measurements. However, some differences in observations between studies were noted. In particular, in young PNG people, increased tissue compressibility was found in the posterior thighs of the infected group using the mechanical Tonometer. In the Myanmar cohort, the between-infection group differences were found using the digital Indurometer at the calf. There may be several reasons for this discrepancy. The PNG cohort had a higher proportion of females (64% vs. 54%) than the Myanmar cohort and a higher mean BMI (19.7 vs. 18.05). In addition, age, gender and hydration were not considered in that analysis. In the current study, the Tonometer did return slightly softer measures in the dominant posterior thigh and non-dominant calf in the Myanmar group, but in this cohort, the differences were not significant. (Table S1). In PNG, no MDA had been available prior to the study, after which treatment was offered to all participants; in Myanmar, MDA had been offered in 2013 and earlier, although less than half of the participants reported taking it. Taken together, these two studies provide the first empirical evidence that there are covert but measurable increases in tissue compressibility and free fluid associated with LF antigenemia, although the optimal site for assessment may differ for different populations. The advance in the current study over that done in PNG was the availability of newer, digital devices and inclusion of age, gender, BMI, and hydration in multivariate regression, which confirmed an independent effect of infection.

The proportion of all infected individuals that will progress to LFRL, while considered to be relatively small, is not well understood. It appears to depend on multiple factors including genetics, geography, exposure to infection, and worm species, and it was not possible in this cross-sectional study to determine which of the positive cases may be at risk of progression to advanced disease, if any. The fact that mean between-infection group differences can be objectively measured suggests that there is an insidious effect of LF antigenemia on skin and subcutaneous tissues in the lower limb, and this is consistent with the current understanding of the pathogenesis of lymphedema [19,20]. Follow-up on this Myanmar cohort may provide some insight into individual variation among antigen-positive persons to define who is most at risk.

The Indurometer gave the clearest indication that tonometry can be used to detect covert lymphatic change in the lower limb. While the Tonometer and SkinFibroMeter may not have detected latent changes in asymptomatic cases in this cohort, their use in assessment of established leg lymphedema from all causes warrants further study. When using these devices to track changes in the same person over time, moderating factors such as age and gender will be immaterial, hydration can be controlled for by administering a drink prior to measurement, and any change in BMI can be considered when interpreting the results, as is already the practice in BCRL. Indurometry and BIS measures may be useful in monitoring clinical progression in people at risk of lower limb lymphedema and may provide an inexpensive means to objectively measure lymphedema in LF populations.

The presence and direction of clinically-relevant changes in the antigen-positive cases in Myanmar support the hypothesis that LF can induce covert changes in the subcutaneous tissues of the lower limbs. This contributes to the case for formal recognition of a Stage 0 in the classification of LF-related lymphedema. The disparity in resources between BCRL and LFRL settings should not be a barrier to transferring reliable and effective protocols for early detection and intervention in lymphedema to LF populations.

## Figures and Tables

**Table 1:** Group characteristics of antigen-positive and antigen-negative participants (positive by either immunochromatographic test (ICT) or Og4C3) at baseline.

	LF Antigen-Positive Cases	LF Antigen-Negative Controls	Mean Diff (95% CI)	*p*=
	*n* = 50	*n* = 48		
Age in years—mean (SD)	15.20 (3.38)	15.48 (3.46)	0.28 (−1.09, 1.07)	0.691 ^a^
Gender				
Female *n* (%)	27 (54%)	28 (58%)		0.410 ^b^
Male *n* (%)	23 (46%)	20 (42%)		0.410 ^b^
Height in cm—mean (SD)	151.80 (12.56)	152.20 (11.52)	0.399 (−4.44, 5.24)	0.870 ^a^
Weight in kg—mean (SD)	42.27 (12.81)	42.30 (10.12)	0.028 (−4.617, 4.670)	0.990 ^a^
BMI in kg/m^2^—mean (SD)	18.05 (3.46)	18.03 (2.65)	−0.012 (−1.239, 1.216)	0.985 ^a^
Body composition *n* = (%)				0.976 ^c^
Median weight	41 (82%)	40 (83%)		
Underweight > −2SD	7 (14%)	6 (13%)		
Overweight > +1SD	2 (4%)	2 (4%)		
Dominant leg right/left	47/3	47/1		0.324 ^b^
Occupation *n* = (%)				0.395 ^c^
Student	14 (28%)	13 (27%)		
Working/other	32/4 (72%)	34/1 (73%)		
Drank liquid *n* = 97				0.590 ^c^
<60 min	13 (26%)	12 (26%)		
>60 min	37 (74%)	35 (74%) (1 NA)		
Consumed 2013 MDA *n* (%)	17 (34%)	22 (46%)		0.383 ^c^

LF = lymphatic filariasis; BMI = body mass index; SD = standard deviation; ^a^ independent samples *t*-test; ^b^ Fishers exact test; ^c^ Pearson chi-square; NA = participant was not asked.

**Table 2:** Between-infection group differences for Indurometer and BIS measures, size, and direction of variation.

Measurement Point Indurometer	Positive *n* = 50	Negative *n* = 48	Mean Difference (%)	Direction in Positive Cases	*p*=
	Mean (SD)	Mean (SD)			
Dominant anterior thigh	4.80 (0.76)	4.72 (0.69)	0.05 (1.1%)	Softer	0.731
Non-dominant anterior thigh	5.10 (0.88)	5.00 (0.69)	0.10 (1.9%)	Softer	0.546
Dominant posterior thigh	4.13 (0.93)	4.06 (0.87)	0.07 (1.7%)	Softer	0.701
Non-dominant posterior thigh	3.88 (0.83)	3.86 (0.95)	0.02 (0.4%)	Softer	0.933
Dominant calf	2.91 (0.57)	2.70 (0.68)	0.21 (7.8%)	Softer	0.096
Non-dominant calf	2.73 (0.65)	2.46 (0.65)	0.27 (11.1%) *^,#^	Softer	0.021
BIS					
Dominant leg *n* = 47/45	2.44 (0.46)	2.56 (0.45)	0.12 (4.9%) ^#^	More fluid	0.220
Non-dominant leg *n* = 46/44	2.62 (0.56)	2.86 (0.59)	0.24 (9.2%) ^#^	More fluid	0.053

SD = standard deviation; * Significant between-group difference *p* ≤ 0.05; ^#^ clinically relevant between-group difference.

**Table 3:** Stepwise regression for moderating factors associated with variation in Indurometer and bio-impedance spectroscopy (BIS) measures.

		Indurometer				BIS	
		Higher Values = Increased Tissue Compressibility				Lower Values = Increased ECF	
		Posterior Thigh B (SE)		Calf B (SE)		Whole Leg B (SE)	
Factor		Dominant	Non−dominant	Dominant	Non−dominant	Dominant	Non−dominant
*Step 1*	*R*^2^=	*0.002*	*0.000*	*0.029*	*0.054*	*0.017*	*0.042*
Antigen Positive		0.070 (0.182)	0.015 (0.180)	0.212 (0.126)	0.272 (0.116) *	−0.117 (0.095)	−0.238 (0.122)
*Step 2*	*R*^2^=	*0.189*	*0.187*	*0.283*	*0.269*	*0.283*	*0.398*
Antigen Positive		0.093 (0.168)	0.049 (0.166)	0.234 (0.111) *	0.286 (0.104) **	−0.108 (0.083)	−0.210 (0.099) *
Gender = Female		0.751 (0.178) **	0.679 (0.175) **	0.639 (0.117) **	0.492 (0.110) **	0.230 (0.087) **	0.485 (0.103) **
Older age		0.022 (0.025)	0.041 (0.025)	0.010 (0.017)	0.024 (0.016)	−0.051 (0.012) **	−0.061 (0.015) **
Underweight		0.136 (0.250)	0.277 (0.247)	−0.052 (0.165)	−0.094 (0.155)	−0.237 (0.120)	−0.302 (0.142) *
Less Recent Hydration		−0.338 (0.177)	−0.223 (0.174)	−0.139 (0.117)	−0.239 (0.110) *	0.107 (0.085)	0.124 (0.101)

ECF = extracellular fluid; SE = standard error; * *p* < 0.05; ** *p* < 0.01.

**Table 4:** Mean values and between-leg differences using the Indurometer and BIS.

	Indurometer (*n* = 98)			BIS (*n* = 90)
	Anterior Thigh	Posterior Thigh	Calf	Whole Leg
Dominant leg mean (SD)	4.74 (0.72)	4.10 (0.90)	2.81 (0.63)	2.50 (0.46)
Non−dominant leg mean (SD)	5.05 (0.79)	3.87 (0.89)	2.60 (0.59)	2.74 (0.59)
Mean difference (SD)	−0.31 (0.31)	0.23 (0.23)	0.21 (0.21)	−0.24 (0.32)
95% CI of the difference	−0.41, −0.21	0.11, 0.35	0.13, 0.28	−0.31, −0.17
% difference	6.5% **	5.6% **	7.5% **	9.6% **^,#^
Direction (dominant leg)	Harder	Softer	Softer	More fluid

SD = standard deviation; ** Significant between-leg difference *p* ≤ 0.01; ^#^ Clinically relevant between-leg difference (tonometry > 10%, BIS > 3%).

## Supplementary Materials

The following are available online at www.mdpi.com/2414-6366/2/4/50/s1, Figure S1: Indurometer, SA Biomedical Engineering; Table S1: Between-infection group differences (independent samples *t*-test) for (a) Digital Indurometer, (b) Mechanical Tonometer, (c) SkinFibroMeter and (d) BIS measures, size and direction of variation; Table S2: Stepwise regression for moderating factors associated with variation in (a) Digital Indurometer, (b) Mechanical Tonometer, (c) SkinFibroMeter and (d) BIS measures; Table S3: Mean values and between-leg differences (paired samples *t*-test) for (a) Digital Indurometer, (b) Mechanical Tonometer, (c) SkinFibroMeter and (d) BIS measures.
