# Supplementary material for: Lymphatic Filariasis Increases Tissue Compressibility and Extracellular Fluid in Lower Limbs of Asymptomatic Young People in Central Myanmar
Source: Trop Med Infect Dis. 2017 Sep 27;2(4):50. doi: 10.3390/tropicalmed2040050 (PMC6082065; doi:10.3390/tropicalmed2040050)
Supplement: Supplementary file 1 [file tropicalmed-02-00050-s001.zip › SI-proof-final/Tables S1, S2, S3.docx]

Supplementary Material

Table S1: Between-infection group differences (independent samples T-test) for a) Digital Indurometer, b) Mechanical Tonometer, c) SkinFibroMeter and d) BIS measures, size and direction of variation

1. Digital Indurometer (higher values indicate higher tissue compressibility/softer tissue)

| **Measurement Point** | **Positive n=50**  Mean (SD) | **Negative n=48**  Mean (SD) | **Mean (%) difference** | **Positive cases are** | **p=** |
| --- | --- | --- | --- | --- | --- |
| **Dominant Anterior Thigh** | 4.77 (0.76) | 4.72 (0.69) | -0.05 (1.1%) | Softer | 0.731 |
| **Non-dominant Anterior Thigh** | 5.10 (0.88) | 5.00 (0.69) | -0.10 (1.9%) | Softer | 0.546 |
| **Dominant Posterior Thigh** | 4.13 (0.93) | 4.06 (0.87) | -0.07 (1.7%) | Softer | 0.701 |
| **Non-dominant Posterior Thigh** | 3.88 (0.83) | 3.86 (0.95) | -0.02 (0.4%) | Softer | 0.933 |
| **Dominant Calf** | 2.91 (0.57) | 2.70 (0.68) | -0.21 (7.8%) | Softer | 0.096 |
| **Non-dominant Calf** | 2.73 (0.65) | 2.46 (0.65) | -0.27 (11.1%)*^#^ | Softer | 0.021 |

1. Mechanical Tonometer (higher values indicate higher tissue compressibility/softer tissue)

| **Measurement Point** | **Positive n=50**  Mean (SD) | **Negative n=48**  Mean (SD) | **Mean (%) difference** | **Positive cases are** | **p=** |
| --- | --- | --- | --- | --- | --- |
| **Dominant Anterior Thigh** | 6.55 (0.92) | 6.55 (0.93) | 0.00^ | Equivocal | 0.998 |
| **Non-dominant Anterior Thigh** | 7.04 (1.09) | 7.09 (0.86) | 0.05 (0.7%) | Equivocal | 0.815 |
| **Dominant Posterior Thigh** | 5.65 (1.16) | 5.57 (1.20) | -0.08 (1.3%) | Softer | 0.753 |
| **Non-dominant Posterior Thigh** | 5.47 (0.95) | 5.57 (1.02) | 0.10 (1.8%) | Harder | 0.629 |
| **Dominant Calf** | 4.29 (0.85) | 4.23 (0.98) | -0.06 (0.3%) | Equivocal | 0.766 |
| **Non-dominant Calf** | 4.18 (0.86) | 3.99 (0.88) | -0.19 (4.8%) | Softer | 0.296 |

^ absolute differences were at the 3rd decimal place therefore meaningful % could not be calculated.

* Significant between-group difference p≤0.05

** Significant between-group difference p≤0.01

# Clinically relevant between-group difference (tonometry >10%, BIS >3%)

Cont….

1. SkinFibroMeter (lower values indicate higher tissue compressibility/softer tissue)

| **Measurement Point** | **Positive n=50**  **Mean N (SD)** | **Negative n=48**  Mean N (SD) | **Mean (%) difference** | **Positive cases are** | **p=** |
| --- | --- | --- | --- | --- | --- |
| **Dominant Anterior Thigh** | 0.08 (0.02) | 0.07 (0.01) | 0.00^ | Equivocal | 0.386 |
| **Non-dominant Anterior Thigh** | 0.07 (0.01) | 0.07 (0.01) | 0.00^ | Equivocal | 0.890 |
| **Dominant Posterior Thigh** | 0.08 (0.02) | 0.08 (0.02) | 0.00^ | Equivocal | 0.917 |
| **Non-dominant Posterior Thigh** | 0.09 (0.02) | 0.08 (0.02) | 0.01^ | Harder | 0.251 |
| **Dominant Calf** | 0.10 (0.04) | 0.10 (0.03) | 0.00^ | Equivocal | 0.524 |
| **Non-dominant Calf** | 0.11 (0.03) | 0.11 (0.03) | 0.00^ | Equivocal | 0.974 |

1. Bio-impedance spectroscopy (Ri:Re, lower values indicate more free fluid)

| **Measurement Point** | **Positive n=50**  **Mean Ri:Re (SD)** | **Negative n=48**  Mean Ri:Re (SD | **% difference** | **Positive cases are** | **p=** |
| --- | --- | --- | --- | --- | --- |
| **Dominant Leg n=47/45** | 2.44 (0.46) | 2.56 (0.45) | 0.12 (4.9%) ^#^ | More fluid | 0.220 |
| **Non-dominant Leg n=46/44** | 2.62 (0.56) | 2.86 (0.59) | 0.24 (9.2%) ^#^ | More fluid | 0.053 |

^ absolute differences were at the 3rd decimal place therefore meaningful % could not be calculated.

* Significant between-group difference p≤0.05

** Significant between-group difference p≤0.01

# Clinically relevant between-group difference (tonometry >10%, BIS >3%)

Table S2: Stepwise regression for moderating factors associated with variation in a) Digital Indurometer, b) Mechanical Tonometer, c) SkinFibroMeter and d) BIS measures

1. Digital Indurometer (higher values indicate higher tissue compressibility)

|  |  | **Anterior Thigh *Β (SE)*** | | **Posterior Thigh *Β (SE)*** | | **Calf *Β (SE)*** | |
| --- | --- | --- | --- | --- | --- | --- | --- |
|  | **Factor** | **Dominant** | **Non-dominant** | **Dominant** | **Non-dominant** | **Dominant** | **Non-dominant** |
| ***Step 1*** | ***R^2^ =*** | *0.001* | *0.004* | *0.002* | *0.000* | *0.029* | *0.054* |
|  | **Antigen Positive** | 0.0514 (0.147) | 0.097 (0.160) | 0.070 (0.182) | 0.015 (0.180) | 0.212 (0.126) | 0.272 (0.116)* |
| ***Step 2*** | ***R^2^ =*** | *0.066* | *0.044* | *0.189* | *0.187* | *0.283* | *0.269* |
|  | **Antigen Positive** | 0.060 (0.146) | 0.104 (0.161) | 0.093 (0.168)^1^ | 0.049 (0.166) | 0.234 (0.111)* | 0.286 (0.104)** |
|  | **Gender = Female** | 0.275 (0.154) | 0.330 (0.170) | 0.751 (0.178)** | 0.679 (0.175)** | 0.639 (0.117)** | 0.492 (0.110)** |
|  | **Older age** | 0.026 (0.022) | -0.012 (0.024) | 0.022 (0.025) | 0.041 (0.025) | 0.010 (0.017) | 0.024 (0.016) |
|  | **Under-weight** | 0.079 (0.216) | 0.085 (0.239) | 0.136 (0.250) | 0.277 (0.247) | -0.052 (0.165) | -0.094 (0.155) |
|  | **Less recent Hydration** | -0.209 (0.153) | -0.095 (0.169) | -0.338 (0.177)^2^ | -0.223 (0.174) | -0.139 (0.117) | -0.239 (0.110)* |

1. Mechanical Tonometer (higher values indicate higher tissue compressibility)

|  |  | **Anterior Thigh *Β (SE)*** | | **Posterior Thigh *Β (SE)*** | | **Calf *Β (SE)*** | |
| --- | --- | --- | --- | --- | --- | --- | --- |
|  | **Factor** | **Dominant** | **Non-dominant** | **Dominant** | **Non-dominant** | **Dominant** | **Non-dominant** |
| ***Step 1*** | ***R^2^ =*** | *0.000* | *0.001* | *0.001* | *0.002* | *0.001* | *0.011* |
|  | **Antigen Positive** | 0.000 (0.187) | -0.046 (0.798) | 0.075 (0.239) | -0.097 (0.199) | 0.055 (0.185) | 0.185 (0.176) |
| ***Step 2*** | ***R^2^ =*** | *0.165* | *0.122* | *0.272* | *0.226* | *0.366* | *0.309* |
|  | **Antigen Positive** | 0.029 (0.175) | -0.016 (0.190) | 0.106 (0.209) | -0.064 (0.180) | 0.093 (0.151) | 0.218 (0.151) |
|  | **Gender = Female** | 0.468 (0.185)* | 0.682 (0.201)** | 1.062 (0.221)** | 0.866 (0.190)** | 1.122 (0.160)** | 0.923 (0.159)** |
|  | **Older age** | 0.071 (0.026) | 0.008 (0.029) | 0.043 (0.031) | 0.036 (0.027) | 0.014 (0023) | 0.030 (0.023) |
|  | **Under-weight** | 0.268 (0.260) | 0.265 (0.283) | 0.554 (0.311) | 0.267 (0.267) | 0.206 (0. 225) | 0.180 (0.224) |
|  | **Less recent Hydration** | -0.292 (0.184) | -0.097 (0.200) | -0.689 (0.220)** | -0.376 (0.189)* | -0.341 (0. 159)* | -0.342 (0.158)* |

* p≤0.05

** p≤0.01

Contd….

1. SkinFibroMeter (lower values indicate higher tissue compressibility)

|  |  | **Anterior Thigh Β (SE)** | | **Posterior Thigh Β (SE)** | | **Calf Β (SE)** | |
| --- | --- | --- | --- | --- | --- | --- | --- |
|  | **Factor** | **Dominant** | **Non-dominant** | **Dominant** | **Non-dominant** | **Dominant** | **Non-dominant** |
| ***Step 1*** | ***R^2^ =*** | *0.008* | *0.000* | *0.000* | *0.014* | *0.004* | *0.000* |
|  | **Antigen Positive** | 0.003 (0.003) | 0.000 (0.003) | 0.000 (0.004) | 0.006 (0.005) | 0.005 (0.007) | 0.000 (0.006) |
| ***Step 2*** | ***R^2^ =*** | *0.238* | *0.192* | *0.263* | *0.191* | *0.226* | *0.153* |
|  | **Antigen Positive** | 0.002 (0.003) | 0.000 (0.002) | -9.027E-5 (0.004) | 0.005 (0.005) | 0.004 (0.007) | -0.001 (0.006) |
|  | **Gender = Female** | -0.014 (0.003)** | -0.012 (0.003)** | -.022 (0.004 | -0.021 (0.005)** | -0.033 (0.007) | -0.022 (0.006) |
|  | **Older age** | -0.001 (0.000) | -2.585E-5 (0.000) | .000 (0.001 | 0.000 (0.001) | 0.000 (0.001) | -0.001 (0.001) |
|  | **Under-weight** | 0.001 (0.004) | -0.002 (0.004) | .004 (0.006 | 0.006 (0.007) | 0.002 (0.010) | 0.009 (0.009) |
|  | **Less recent Hydration** | 0.007 (0.003)* | 0.002 (0.003) | .008 (0.004 | 0.004 (0.005) | 0.019 (0.007) | 0.013 (0.006) |

1. Bio-impedance spectroscopy (SBF7) (lower values indicate higher free fluid)

|  |  | **Whole Leg Β (SE)** | |
| --- | --- | --- | --- |
|  | **Factor** | **Dominant Leg** | **Non-dominant Leg** |
| *Step 1* | *R^2^ =* | *0.017* | *0.042* |
|  | **Antigen Positive** | -0.117 (0.095) | -0.238 (0.122) |
| *Step 2* | *R^2^ =* | *0.283* | *0.398* |
|  | **Antigen Positive** | -0.108 (0.083) | -0.210 (0.099)* |
|  | **Gender = Female** | 0.230 (0.087)** | 0.485 (0.103)** |
|  | **Older age** | -0.051 (0.012)** | -0.061 (0.015)** |
|  | **Under-weight** | -0.237 (0.120) | -0.302 (0.142)* |
|  | **Less recent Hydration** | 0.107 (0.085) | 0.124 (0.101) |

* p≤0.05

** p≤0.01

Table S3: Mean values and between-leg differences (paired samples t-test) for a) Digital Indurometer, b) Mechanical Tonometer, c) SkinFibroMeter and d) BIS measures

1. Digital Indurometer n=98 (higher values indicate higher tissue compressibility)

|  | **Dominant leg**  **Mean (SD)** | **Non-dominant leg**  **Mean (SD)** | **Mean difference (SD)** | **95% CI of the difference** | **% difference** | **Direction of variation**  **Dominant leg** | **p=** |
| --- | --- | --- | --- | --- | --- | --- | --- |
| **Anterior Thigh** | 4.74 (0.72) | 5.05 (0.79) | -0.31 (0.31) | -0.41, -0.21 | 6.5%** | Harder | p<0.001 |
| **Posterior Thigh** | 4.10 (0.90) | 3.87 (0.89) | 0.23 (0.23) | 0.11, 0.35 | 5.9%** | Softer | p<0.001 |
| **Calf** | 2.81 (0.63) | 2.60 (0.59) | 0.21 (0.21) | 0.13, 0.28 | 7.9%** | Softer | p<0.001 |

1. Mechanical Tonometer n=98 (higher values indicate higher tissue compressibility)

|  | **Dominant leg**  **Mean (SD)** | **Non-dominant leg**  **Mean (SD)** | **Mean difference (SD)** | **95% CI of the difference** | **% difference** | **Direction of variation**  **Dominant leg** | **p=** |
| --- | --- | --- | --- | --- | --- | --- | --- |
| **Anterior Thigh** | 6.55 (0.92) | 7.06 (0.98) | -0.52 (0.76) | -0.67, -0.36 | 7.9%** | Harder | p<0.001 |
| **Posterior Thigh** | 5.61 (1.18) | 5.52 (0.98) | 0.09 (0.80) | -0.07, 0.25 | 1.6% | Softer | 0.273 |
| **Calf** | 4.26 (0.91) | 4.09 (0.87) | 0.18 (0.56) | 0.06, 0.29 | 4.1%** | Softer | 0.002 |

1. SkinFibroMeter n=98 (lower values indicate higher tissue compressibility)

|  | **Dominant leg**  **Mean (SD)** | **Non-dominant leg**  **Mean (SD)** | **Mean difference (SD)** | **95% CI of the difference** | **% difference** | **Direction of variation**  **Dominant leg** | **p=** |
| --- | --- | --- | --- | --- | --- | --- | --- |
| **Anterior Thigh** | 0.07 (0.02) | 0.07 (0.01) | 0.01 (0.01) | 0.00, 0.01 | 8.8%** | Harder | p<0.001 |
| **Posterior Thigh** | 0.08 (0.02) | 0.09 (0.02) | -0.00 (0.02) | -0.01, 0.00 | 1.9% | Softer | 0.424 |
| **Calf** | 0.10 (0.04) | 0.11 (0.03) | -0.01 (0.03) | -0.01, -0.00 | 7.2%* | Softer | 0.014 |

1. Bio-impedance spectroscopy (SBF7) n=90 (lower values indicate higher free fluid)

|  | **Dominant leg**  **Mean (SD)** | **Non-dominant leg**  **Mean (SD)** | **Mean difference (SD)** | **95% CI of the difference** | **% difference** | **Direction of variation**  **Dominant leg** | **p=** |
| --- | --- | --- | --- | --- | --- | --- | --- |
| **Whole Leg** | 2.50 (0.46) | 2.74 (0.59) | -0.24 (-0.32) | -0.31, -0.17 | 9.6%**^#^ | More fluid | p<0.001 |

* Significant between-leg difference p≤0.05

** Significant between-leg difference p≤0.01

# Clinically relevant between-leg difference (tonometry >10%, BIS >3%)
