# Supplementary figures and images for: Lymphatic Filariasis Increases Tissue Compressibility and Extracellular Fluid in Lower Limbs of Asymptomatic Young People in Central Myanmar
Source: Trop Med Infect Dis. 2017 Sep 27;2(4):50. doi: 10.3390/tropicalmed2040050 (PMC6082065; doi:10.3390/tropicalmed2040050)

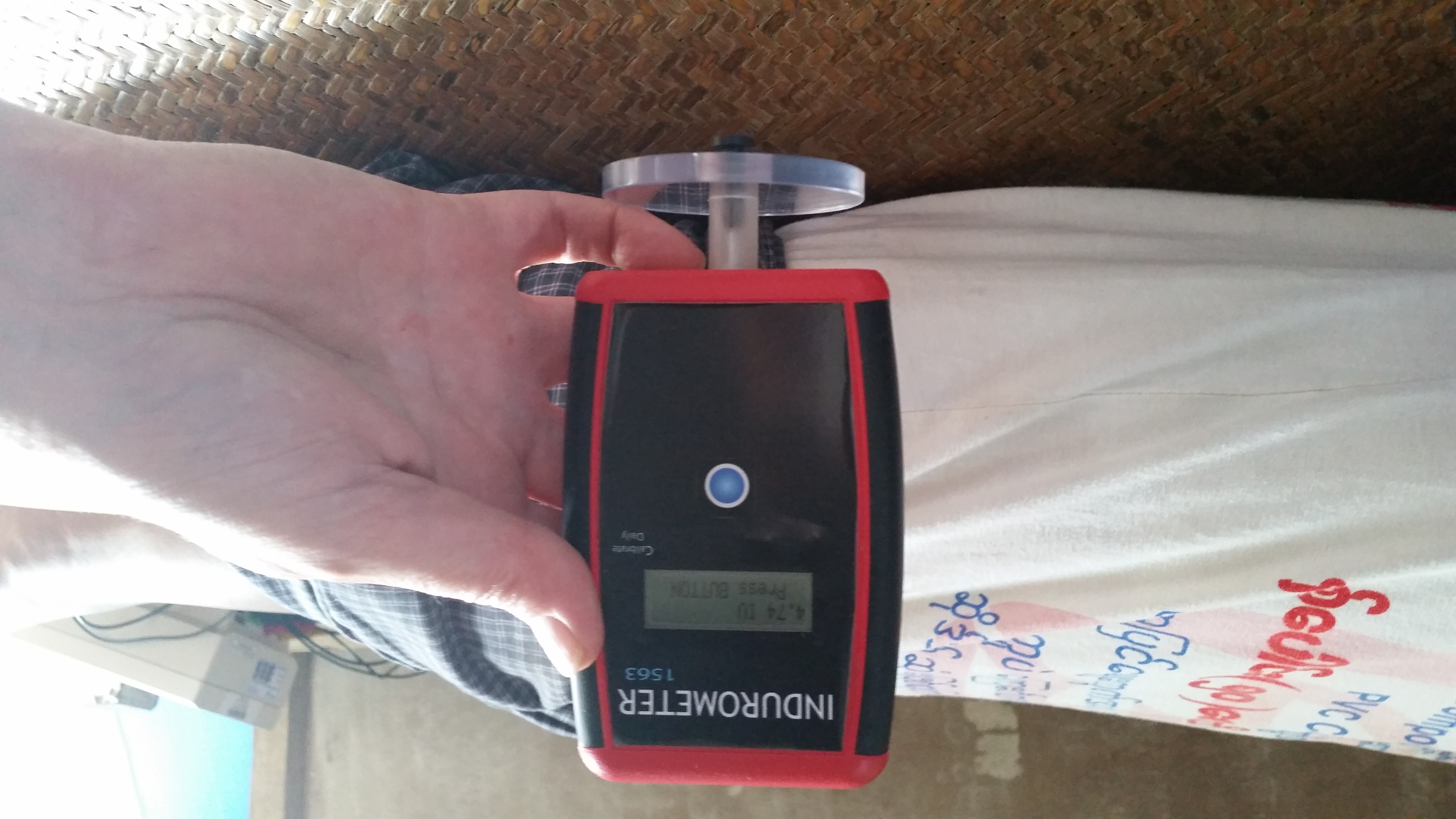

Supplement: Supplementary file 1 [file tropicalmed-02-00050-s001.zip › SI-proof-final/Figure S1.jpg]
